# Atypical responses of a large catchment river to the Holocene sea-level highstand: The Murray River, Australia

**DOI:** 10.1038/s41598-020-61800-x

**Published:** 2020-05-05

**Authors:** Anna M. Helfensdorfer, Hannah E. Power, Thomas C. T. Hubble

**Affiliations:** 1 School of Geosciences, The University of Sydney, Sydney, NSW 2006 Australia; 2 School of Environmental and Life Sciences, The University of Newcastle, Callaghan, NSW 2308 Australia

**Keywords:** Palaeoclimate, Environmental sciences

## Abstract

Three-dimensional numerical modelling of the marine and fluvial dynamics of the lower Murray River demonstrate that the mid-Holocene sea-level highstand generated an extensive central basin environment extending at least 140 kilometres upstream from the river mouth and occupying the entire one to three kilometre width of the Murray Gorge. This unusually extensive, extremely low-gradient backwater environment generated by the two metre sea-level highstand captured most, if not all, of the fine-grained sediment discharged from the 1.06 million square kilometre Murray-Darling catchment. This material was sequestered within a >60 kilometre long, >10 metre thick valley-wide deposit of finely laminated mud. This previously unrecognised sediment trap persisted from 8,518 to 5,067 cal yr BP preventing sediment delivery to the marine environment. Its identification requires that mid-Holocene climate reconstructions for southeastern Australia based on fluctuations in the delivery of fine-grained sediment to the ocean offshore the lower Murray River’s mouth must be re-evaluated.

## Introduction

**Figure 1:**
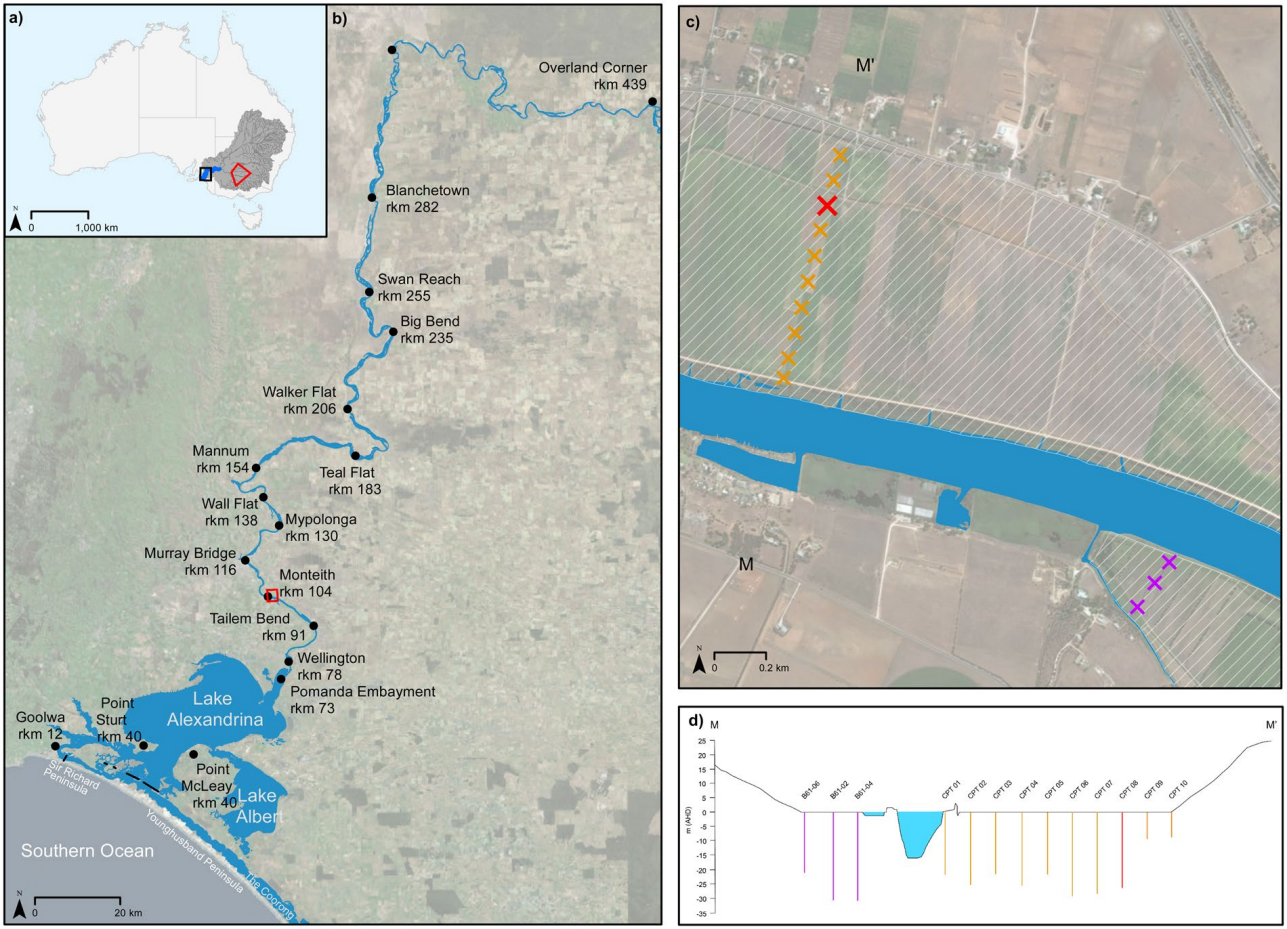
Overview of study site. (**a**) The Murray-Darling Basin (grey) drains the twin Murray and Darling catchments, whose major watercourses are shown in dark grey. The confluence of the Murray and Darling Rivers marks the upstream extent of the lower Murray River (blue) which flows to the Southern Ocean in South Australia. The extent of the Riverine Plain is given in red (see Discussion for context). (**b**) The lower Murray River enters the Murray Gorge at Overland Corner (rkm 439) and remains confined until debouching into Lake Alexandrina at Wellington (rkm 78). The modern-day estuary comprises the Lower Lakes, Alexandrina and Albert, together with the Coorong lagoon. The lower Murray River flows through the Murray Mouth into the Southern Ocean between Younghusband and Sir Richard Peninsulas. A series of five barrages (black) regulate saline incursion into the estuary. (**c**) Fieldwork was conducted at Monteith (rkm 104) on grazing land comprising a reclaimed backswamp (white hatch) situated behind an artificial levee on the left bank of the river. Ten CPTs were taken at 100 m spacing commencing 100 m from the channel (orange crosses), with core Monteith-A extruded at the same site as CPT08 (red cross). Three CPTs collected as part of an alternate study completes the transect on the right bank of the river (purple crosses). (**d**) Elevation profile of transect M-M’ showing the position of each CPT relative to the width of the Murray Gorge. CPTs were pushed until refusal with penetration depths reaching beyond 20 m in all but the two at the valley margins. Satellite imagery source: Esri.

Effective natural resource management benefits from a thorough understanding of how a system functioned prior to anthropogenic modification. Palaeo-climatic data are often used to inform natural resource management, with sequences of Holocene sediments providing a record that constrains a system’s predicted response to a changing climate and sea level^1^. The political, economic and environmental ramifications of natural resource allocation decisions will become increasingly contentious in coming decades as the consequences of a changing climate become more apparent^1,2^. Managers will become increasingly reliant on high quality palaeo-climatic data to inform their policies^1,2^. This is particularly the case for intensively managed river systems, such as Australia’s Murray-Darling Basin (MDB), that support large-scale agriculture whilst also being important ecological refuges (Fig. 1). The MDB comprises the Murray and Darling sub-catchments which drain over 1 million km^2^ and is Australia’s most economically important agricultural region. At the terminus of the MDB, the lower Murray River (LMR) is confined within a bedrock valley and entrenched within the Tertiary limestone of the Murray Gorge (Fig. 1). Here, the valley fill comprises sediments of a single cycle of lowstand, transgression and highstand only. The LMR debouches into Lake Alexandrina and then flows through the Murray Mouth to the Southern Ocean (Fig. 1). The Murray’s barrier estuary developed in response to a rapidly rising sea level during the Holocene with the formation of Sir Richard and Younghusband peninsulas and the development of the central basin lakes Alexandrina and Albert^3,4^ (Fig. 1).

The MDB has been increasingly managed since 1900 to accommodate the competing needs of irrigation and drinking water supply for development while maintaining environmental flows in a hydroclimate that is prone to long-term droughts^1^. Given the challenges of a warming and drying climate^5^ the successful management of this system and its water allocation policy will be informed by an improved understanding of southeastern Australia’s Holocene climate.

The Holocene climate of southeastern Australia is typically characterised by wetter than present-day conditions during the early- to mid-Holocene, before a shift to increased climatic variability and an overall trend to aridity in the late-Holocene^6–8^. Within the MDB, approximately 90% of flow is derived from the Murray River and its tributaries^9^, with the annual snow melt on the southeast Australian highlands producing significant seasonal flow variability within this sub-catchment. Murray sub-catchment annual rainfall and flow variability is currently dominated by the El Niño-Southern Oscillation (ENSO), which generates inter-annual variability, and the Pacific Decadal Oscillation (PDO), which drives decadal to centurial variability^2,8,10^. The semi-arid Darling sub-catchment receives its most significant flows from the Inter-Tropical Convergence Zone (ITCZ) summer monsoon^2,8,10^. Analyses of a record of uninterrupted sediment deposition preserved in marine cores MD03-2611 and MD03-2607, taken offshore from the Murray’s Mouth on the Lacepede Shelf, have been used to generate the only available and commonly cited palaeo-climatic reconstruction derived from sediment captured from the entire MDB^2,6,11^. Analyses of the terrigenous sediment flux within these cores led Gingele *et al*^6,11^. to conclude that, except for a brief dry period from 9,000–8,000 yr BP, humid conditions prevailed throughout the early- to mid-Holocene. This was followed by a shift to increasingly arid conditions reaching conditions similar to those of the present-day by 5,500 yr BP^6,11^.

As sea level rose rapidly in the early-Holocene, river valleys were drowned forming the precursors of the modern-day estuaries and the river valleys of southeastern Australia’s stable craton^12,13^. Increased availability of marine sediment saw the development of the Murray estuary’s barrier complex from approximately 8,000 yr BP, prior to the +2 m Holocene highstand at 7,000–6,000 yr BP^3,12,14–16^. Worldwide, landward migration of fluvial, estuarine, and marine environments caused a continuing decrease in the depositional gradient of coastal plain rivers which typically resulted in the deposition of an upward fining sequence within fluvial deposits in coastal incised valleys^17^. This commonly presents as a transition from high-energy fluvial sands to low-energy mud-dominated sediments^17^. This is also evident in the LMR where the valley-fill transitions from the braid plain sands of the Monoman Formation to the low-energy clays and silts of the Coonambidgal Formation^18^. With their ample accommodation space, young estuaries were very efficient sediment traps, which sequestered terrigenous and marine sediment as they infilled^12,19,20^. The estuarine fill in the main body of Lake Alexandrina is characterised by a laminated silt-clay central basin deposit, known as the St Kilda Formation, which began accumulating by at least 8,000 yr BP and was well-established and regionally extensive by 5,500 yr BP^21,22^.

A previous assessment of the extent of the palaeo-Murray estuary demonstrated that the +2 m higher-than-present sea level of the mid-Holocene highstand generated an estuarine environment throughout the Lower Lakes and well upstream into the LMR^23^. The rapid rise in sea level inundated the entire width of the several kilometre-wide Murray Gorge and extended upstream at least to Blanchetown (river kilometre (rkm) 282), creating a single, continuous, body of water quite unlike the present-day channel and fringing swamps^23^. At highstand, the flooded Murray Gorge presented an extensive backwater zone with an enlarged central basin environment that occupied Lake Alexandrina and the lower reaches of the LMR at least as far upstream as Monteith (rkm 104) and possibly upstream to Walker Flat (rkm 206)^23^. Conventional models of estuarine facies designation place the upstream limit of the central basin at the point where the river debouches into the lagoon, with the tidal limit typically only propagating tens of kilometres beyond this point^17,20^. The immense longitudinal extent of the Murray estuary’s depositional response is atypical and presents an end-member example of an extremely low gradient coastal plain system.

Here, we apply a novel, multidisciplinary approach to evaluate the findings of 3D hydrodynamic modelling of the Murray estuary during the Holocene highstand^23^ against a well dated core and sediment data to understand the Holocene geomorphologic evolution of the LMR and Murray estuary. Specifically, we:

- Establish the lateral extent of the water body that occupied the Murray Gorge at the Holocene highstand by correlating thirteen closely spaced cone penetrometer soundings, taken along a transect perpendicular to the modern-day channel, with an undisturbed 30 m sediment core (Monteith-A) taken at Monteith, 104 rkm upstream of the Murray Mouth;
- Conduct a sedimentary analysis on core Monteith-A, with analyses for grainsize, moisture content, bulk density, total organic carbon, and radiocarbon dating (to establish a chronology and sedimentation rates), to determine sedimentary units and assign facies designation constraining the timing and nature of geomorphologic evolution;
- Further confirm Helfensdorfer *et al*.’s^23^ best-estimate Holocene highstand and pre-anthropogenic 2D hydrodynamic model in 3D to resolve the potential influence of estuarine stratification; and
- Assess to what extent the Murray estuary propagated upstream into the confines of the Murray Gorge at the Holocene highstand and independently verify, through the combination of 3D modelling and sedimentology, the conclusion of Helfensdorfer *et al*^23^. that the palaeo-Murray’s central basin, characterised by a laminated silt-clay sequence as described by Barnett^21,22^, extended from Lake Alexandrina at least as far upstream as Monteith (rkm 104).

## Results

### Hydrodynamic modelling

**Figure 2:**
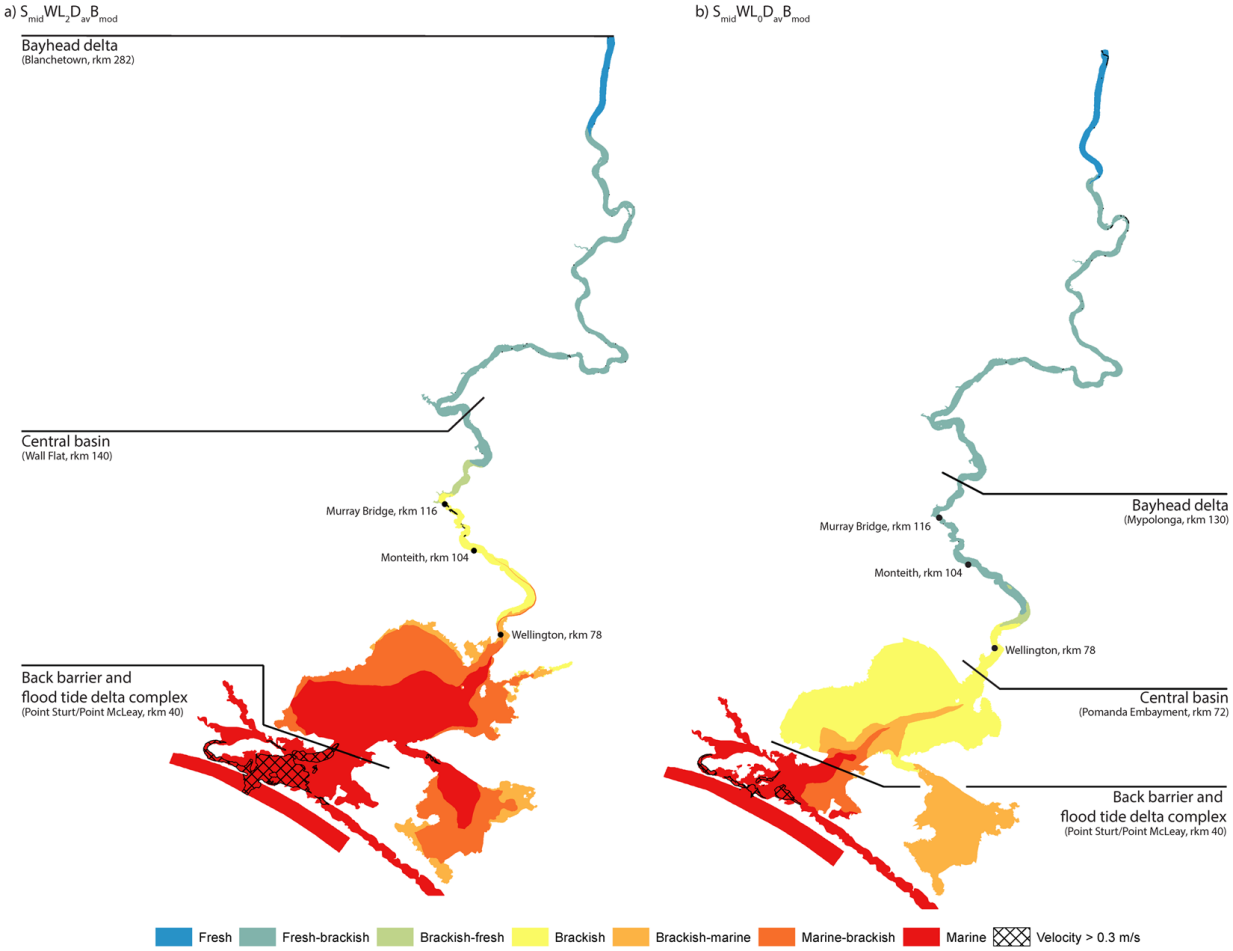
Modelled maximum salinity and velocity magnitude and inferred resulting morphological zonation. (**a**) 3D depth-averaged maximum salinity reached from a best-estimate Holocene highstand scenario (S_mid_WL_2_D_av_B_mod_) demonstrates that the Lower Lakes were subject to significant marine incursion driving the brackish limit beyond Murray Bridge (rkm 118). The flood-tide delta is the only region where maximum velocity magnitudes exceed the limit for deposition of a laminated sequence over significant areas^24–26^. (**b**) The reduction of sea level in the late Holocene (modelled as scenario S_mid_WL_0_D_av_B_mod_) causes a significant change in the palaeo-environmental character of the region restricting the brackish limit to Wellington (rkm 85) and supressing marine incursion to the flood tide delta. Salinity categorisation is based on the classification scheme of Tooley^64^.

A best-estimate 3D Holocene highstand scenario (model scenario code: S_mid_WL_2_D_av_B_mod_, see methodology) supports and extends the 2D model results of Helfensdorfer *et al*.^23^, which showed that the +2 m sea level of the Holocene highstand generated an estuarine palaeo-environment throughout the Lower Lakes and upstream into the lower reaches of the LMR (Fig. 2a). This high-resolution 3D model suggests that the lower Murray Gorge flooded completely, with the depth-averaged marine-brackish (10 psu) limit penetrating upstream as far as Tailem Bend (rkm 91; Fig. 2a). The tidal limit extended beyond the model extent (minimum Blanchetown, rkm 282; Fig. 2a). A central basin environment occupied the region upstream of Point Sturt and Point McLeay (rkm 40) up into the Murray Gorge to Wall Flat (rkm 140; Fig. 2a), correlating well with the median extent given by the suite of 2D models in Helfensdorfer *et al*^23^. (median Mannum, rkm 147). At highstand, maximum flow velocities were <0.3 m/s for 95% of the model domain, consistent with generating an environment conducive to the deposition of a laminated silt-clay sequence^24–26^. Significant areas subject to velocities exceeding 0.3 m/s were only extant seaward of Point Sturt/Point McLeay (rkm 40, within the flood-tide delta; Fig. 2a).

Estuarine infill and a decline in sea level to present-day levels, representative of modern pre-modification conditions (model scenario code: S_up_WL_0_D_av_B_mod_, see methodology), significantly reduced saline incursion to the Lower Lakes and limited marine influence to the flood-tide delta region, with the brackish limit propagating only as far upstream as Wellington (rkm 85; Fig. 2b). Under these conditions, the central basin is inferred to be restricted to the Lower Lakes with the upstream limit remaining within the upper reaches of Lake Alexandrina at the Pomanda Embayment (rkm 72; Fig. 2b). Further, maximum flow velocities are reduced, enabling conditions suitable for the deposition of a laminated silt-clay sequence throughout almost the entire model domain (99%; Fig. 2b).

### Analyses of core Monteith-A

#### Chronology

**Figure 3:**
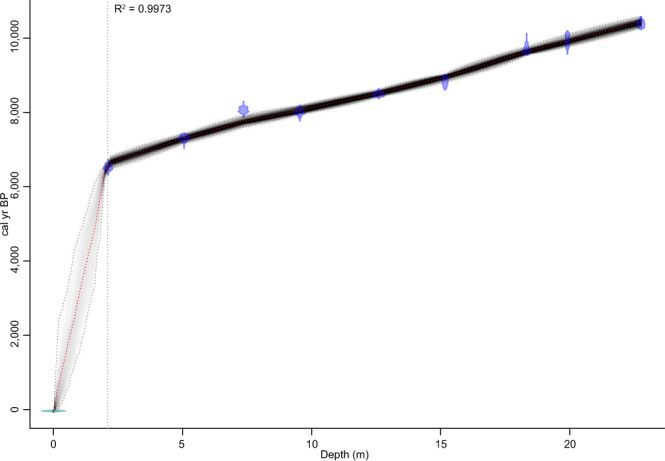
Bacon age-depth model produced from core Monteith-A dates. The model is very well constrained for the period between the youngest and oldest dates (6,513–10,374 yr BP; R^2^ = 0.997), giving an average sediment accumulation rate of 0.60 cm/yr for this 20.65 m range. Assuming the surface of the core is modern, the model gives an average sediment accumulation rate of 0.03 cm/yr for the top 2.10 m. The model returns all calibrated ^14^C dates (purple), the ‘best’ model based on the mean age for each depth (red dashed line) and the 95% confidence interval (grey dashed lines), with the degree of shading between the 95% confidence intervals representing the likelihood of the calendar age.

**Figure 4:**
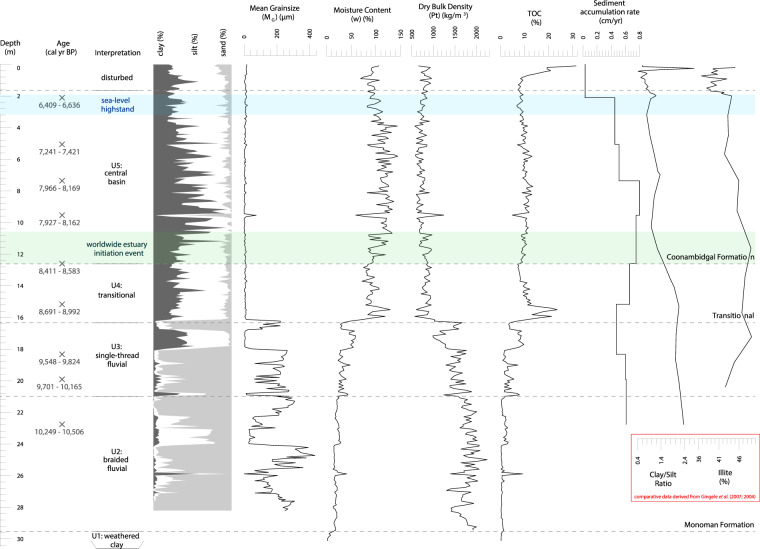
Sedimentary analysis of core Monteith-A relative to sea level and key proxies from offshore marine cores MD03-2611 and MD03-2607. Calibrated ages together with the grainsize distribution, moisture content, dry bulk density, TOC and sediment accumulation rate led to the interpretation of five units: basal weathered clay (Unit 1), lowstand braided fluvial faces (Unit 2), transgressive single-thread fluvial (Unit 3), transitional fluvial bayhead delta (Unit 4) and central basin facies (Unit 5). The top 1.65 m is potentially disturbed due to agricultural activities at the site. Fluctuations in the clay/silt ratio and illite percentage are given for offshore marine cores MD03-2607 and MD03-2611 respectively^6,11^. The timing of the Holocene sea-level highstand (7,000–6,000 yr BP) is demarcated by blue shading^15,16^, and the 8,500–8,200 yr BP worldwide estuarine initiation event by green shading^13,27,35,36^.

The chronology for core Monteith-A was determined with five ^14^C radiocarbon dates with ages that span the early- to mid-Holocene from 10,249–10,506 cal yr BP to 6,509–6,636 cal yr BP (Table 1). These dates present an increasing age with depth, with two ages 2.18 m apart (at 7.36 m and 9.54 m) returning near identical calibrated ages of 7,966–8,169 and 7,927–8,162 cal yr BP respectively (Table 1). Assuming the surface is modern, the top 2.10 m (above the youngest date) has a markedly different sediment accumulation rate of 0.03 cm/yr (Fig. 3). Given the potential for disturbance of the near surface by agricultural activities, this top-most 2.1 m section of the core was excluded from further chronological analysis. For the period spanning the five radiocarbon ages (20.65 m), the age-depth model is very well constrained with a linear regression coefficient of R^2^ = 0.997, such that depth in the core is considered to be a valid approximation of age (Fig. 3). The model returns a mean sediment accumulation rate of 0.60 cm/yr (Fig. 3), with minor variations from the mean rate that correspond to the rise of sea level during the period of deposition. The period 8,516–7,750 cal yr BP marks the most rapid sediment accumulation in the core, with an average of 0.77 cm/yr, and is likely a consequence of the rapid rise in sea level caused by the melting of the Laurentide ice sheet at approx. 8,200 yr BP (Fig. 4)^13,27^. The worldwide initiation of Holocene estuaries has been attributed to this event and our chronology demonstrates that the response of the LMR is consistent with the world’s other major river systems^13,27^. Sediment accumulation rates are slowest at highstand with an average of 0.48 cm/yr between 7,750–6,543 cal yr BP (Fig. 4).

**Table 1:** Conventional and calibrated ages for core Monteith-A ^14^C samples.

Lab ID	Depth (m)	^14^C date (yr BP ± 1 σ)	Material	Calibrated age (2 σ) (cal. yr BP)	Probability (%)	Median calibrated age (2 σ) (cal. yr BP)
UB-38709	2.11	5,761 ± 43	>63 µm charcoal fragments	6,409–6,636	100	6,513
UBA-38326	5.06	6,413 ± 45	>63 µm charcoal fragments	7,241–7,421 7,178–7,214	93.9 6.1	7,308
UBA-38765	7.36	7,282 ± 49	>63 µm charcoal fragments	7,966–8,169	100	8,062
UBA-38710	9.54	7,221 ± 58	fibrous organic fragment	7,927–8,162 7,871–7,895	96.0 4.0	7,998
UBA-38325	12.60	7,748 ± 44	>63 µm charcoal fragments	8,411–8,583	100	8,490
UBA-36739	15.17	8,006 ± 37	charred fibrous organic fragment	8,691–8,992 8,649–8,678 8,683–8,689	93.6 5.4 1.0	8,839
UBA-38766	18.33	8,757 ± 44	>63 µm charcoal fragments	9,548–9,824 9,843–9,869 9,872–9,887 9,827–9,831	94.1 3.4 2.0 0.5	9,671
UBA-38186	19.91	8,884 ± ± 54	charred fibrous organic fragment	9,701–10,165	100	9,929
UBA-38187	22.76	9,256 ± 44	charred fibrous organic fragment	10,249–10,506	100	10,374

Nine charcoal or fibrous organic samples were taken from the top 23 m of core and analysed at lab UBA. Calibrated ages with the greatest probability are referred herein.

#### Sediment grainsize and characteristics

High resolution optical imagery and radiographs of the Holocene sediments of core Monteith-A (up to 24.12 m) are presented in Fig. S1. The weathered claystone basal unit (Unit 1; 30.12–29.52 m) underlies the Monoman Formation, which, in this core, presents as two distinct units: unit 2 and unit 3. Unit 2 (29.52–20.99 m) is comprised of fine and medium sands with an average mean grainsize of 186.6 µm and a clay:silt:sand (C:S:S) ratio of 3:18:79 (Fig. 4). The boundary between Units 2 and 3 (20.99 m; 10,112 cal yr BP) is marked by an abrupt change from unsorted grey (7.5Y 5/1) sands to banded finer greenish grey (5G 5/1) to coarser grey (7.5Y 5/1) sands (Fig. S1) and a sharp increase in moisture content and total organic carbon (TOC; Fig. 4). Unit 3 (20.99–16.34 m) presents a decrease in average mean grainsize to 136.6 µm and a C:S:S of 14:23:63 (Fig. 4). The two sand units also differ in their physical properties with an increase in average moisture content and TOC and decrease in average dry bulk density from 20 to 36%, 1.21 to 4.36% and 1,775 to 1,448 kg/m^3^ between Units 2 and 3 respectively (Fig. 4). A 3.73 m thick transitional sequence (16.34–12.61 m) comprising mottled fine and medium silts (Unit 4) separates the Monoman Formation sands and the Coonambidgal Formation muds with its basal boundary (9,200 cal yr BP) marked by a distinct colour and textural change (Fig. S1). Unit 4 has an average mean grainsize of 19.1 µm and a C:S:S of 30:59:11, which differs significantly from the grainsize parameters of Unit 3 signalling an abrupt change in depositional conditions (Fig. 4). The transition out of this mottled grey (7.5Y 5/1), greenish grey (5G 5/1) and dark greenish grey (5G 3/1) sequence is gradational in colour and texture but presents a small incremental increase in TOC and moisture content and decrease in dry bulk density at 12.61 m (8,518 cal yr BP; Fig. 4) which we interpret as a subtle shift in depositional condition demarcating the limit of Unit 4. Units 4 and 5 differ in average moisture content, TOC and dry bulk density by 90 to 110%, 11.33 to 10.00% and 838 to 738 kg/m^3^ respectively. Unit 5 presents alternating 0.5 to 2 mm thick dark-coloured grey (7.5Y 4/1) laminations comprising fine to medium silts (average grainsize of 8.6 µm and C:S:S of 31:61:8) and light-coloured greenish grey (5G 6/1) laminations comprised of clay to very fine silt (average grainsize of 1.8 µm and C:S:S of 65:33:2). Moisture content, dry bulk density and TOC averages of 104%, 766 kg/m^3^ and 10% respectively, with the significant increase in TOC in the near surface material (0–0.7 m) due to the presence of roots (Fig. 4).

### Cone penetrometer soundings profile

Interpretation of cone penetrometer test (CPT) results using Robertson’s^28^ soil behaviour type (SBT) identifies a clear distinction between two sedimentary sequences in all CPTs beyond the valley margins (i.e. with the exception of B61-06, CPT09 and CPT10): an upper sequence comprising clays and sensitive fine-grained sediments (Fig. S2 clusters C1-C2), and an underlying sequence comprising silts and sands (Fig. S2 clusters M1-M4). This cross-valley uniformity demonstrates a valley-wide transition from the coarse-grained Monoman Formation to the fine-grained Coonambidgal Formation which is consistent with previous accounts of the Murray Gorge’s valley fill^18,29^. At the study site, this near horizontal transition from lower to upper valley fill occurs at a depth of approximately 14–19 m across the 1,200 m width of the drowned river valley, which is reflected in the mean grainsize of Units 2 and 3 when compared with Units 4 and 5 within core Monteith-A (Fig. 4).

**Figure 5:**
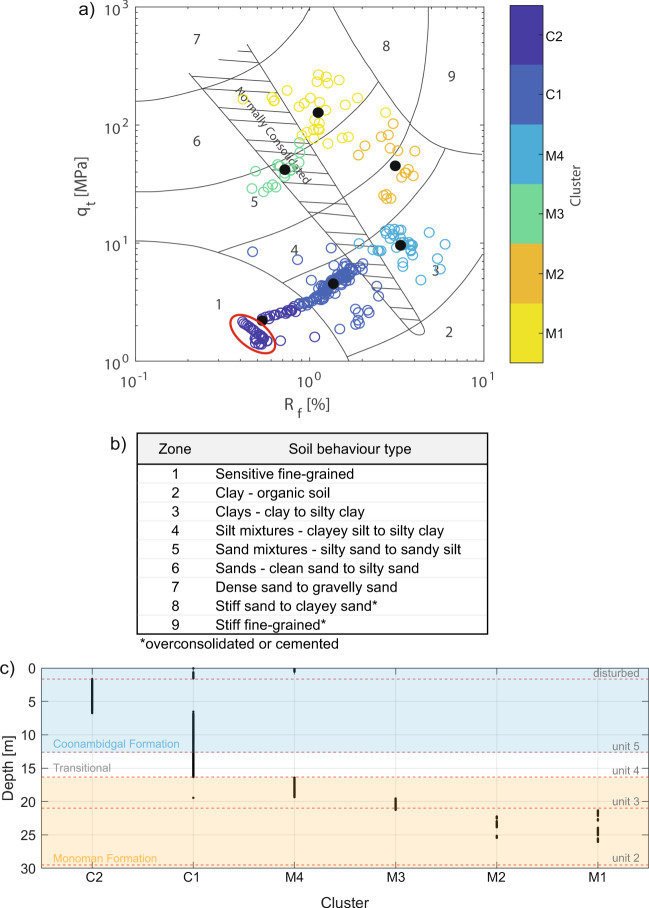
Correlation of facies identified in core Monteith-A to results of CPT08 applying Robertson’s^28^ SBT. (**a**) Plotting CPT08 on Robertson’s^28^ *q*_*c*_/*R*_*f*_ plot and performing a k-medoids clustering analysis reveals six distinct clusters, with sediments of the Monoman Formation identified as clusters M1-M4 and sediments of the Coonambidgal Formation identified as clusters C1-C2. A sub-cluster within cluster C1 (circled in red) comprises drought-affected surficial and shallow sediment. (**b**) Robertson’s^28^ SBT provides a description of sediment based on nine zones distinguished through CPT results *q*_*c*_ and *R*_*f*_. (**c**) Plotting clusters relative to depth facilitates comparison with sedimentary units identified within core Monteith-A (red dashed lines). Sedimentary unit boundaries within core Monteith-A correlate well with the clustering analysis of *q*_*c*_/*R*_*f*_ for CPT08.

This simple division between the Monoman and Coonambidgal Formations has been confirmed and further extrapolated to identify the sedimentary units across the valley through a correlation with core Monteith-A. A k-medoids clustering analysis^30^ of Robertson’s^28^ SBT data obtained from the CPT collected adjacent to the sediment core (CPT08) identifies six clusters (see methodology) which, when plotted relative to depth, allows clusters to be linked to the facies identified in core Monteith-A (Fig. 5). Unit 2 presents in CPT08 as silt mixtures to sands (clusters M1 and M2) with the sequence boundary to Unit 3 correctly placed (Fig. 5). The distinction between the lowstand and transgressive sand facies (Unit 2 to 3) is marked by a reduction in consolidation (Fig. 5); however, the clustering analysis suggests Unit 3 is better represented as two distinct units (clusters M3 and M4), with a sequence boundary at −19.5 m (9,844 cal yr BP). We interpret this division, which was not identified through analysis of core Monteith-A, as illustrating the highly transitional nature of the transgressive system during this period. The boundary between Units 3 and 4 is also correctly placed at −16.34 m (clusters M4 and C1; Fig. 5), however, a limitation is that the clustering analysis cannot distinguish between Units 4 and 5 (cluster C1; Fig. 5a), with our division identified by a subtle change in physical properties at −12.61 m (Fig. 4) which correlates well with the division between SBT 1 and 3 (Fig. 5a). The clustering analysis suggests a division at −6.3 m, which is not reflected through the analysis of core Monteith-A. Rather, we suggest that this is an artefact of drying during the Millennium drought (1997–2011) when the significant lowering of the water table caused clays of the reclaimed swamps, such as those at the study site, to crack to depths in excess of 3.5 m^31^. This is consistent with the maximum depth of the sub-cluster (circled in Fig. 5a) of 4.35 m. Overall, the correlation of CPT08 with core Monteith-A confirms that the division of units assigned based on the visual log (Fig. S1) and sedimentary data (Fig. 4) can also be identified by the geotechnical properties of the sediment (Fig. 5). This calibration validates the use of CPT soundings to extrapolate the sedimentary units identified in core Monteith-A across the width of the valley.

**Figure 6:**
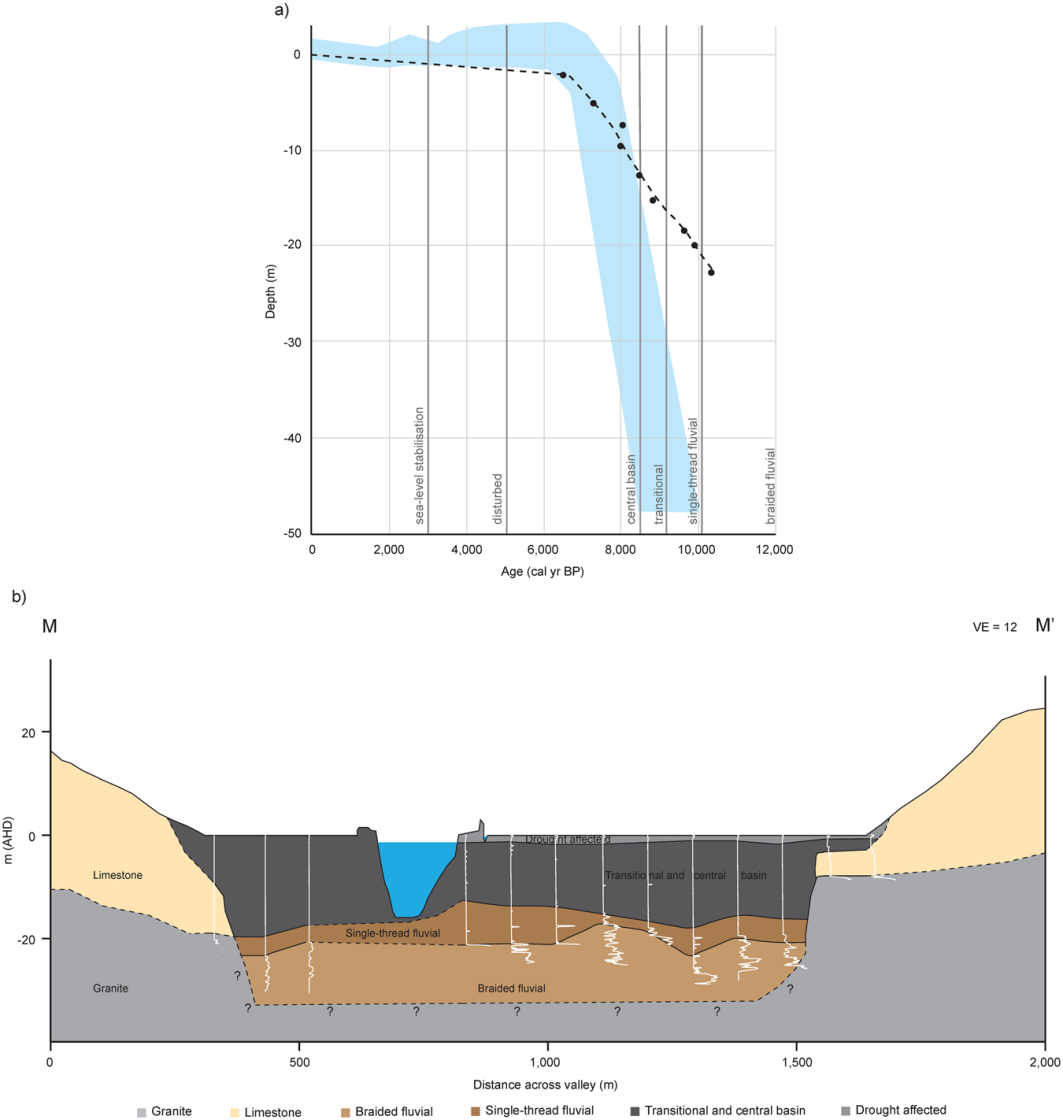
Core Monteith-A sedimentary facies relative to sea level and valley-wide cross section extrapolated from CPTs collected in transect. (**a**) The Bacon age-depth plot for core Monteith-A is plotted, relative to facies designation, against the Holocene sea-level envelope for the Southern Australian coast (blue)^15^. The inception of the Murray estuary, and commencement of deposition of the central basin sequence, is synchronous with the onset of marine flooding at this location. Calibrated ^14^C ages are denoted by black points, with the approximate timing of sea-level stabilisation to the present-day level shown as 3,500 yr BP^4,44–46^. (**b**) A valley-wide cross section at Monteith (rkm 104; transect M-M’ given in Fig. 1c,d) developed from SBT analysis of thirteen CPTs reveals that the central basin (Unit 5) is uninterrupted across the whole width of the valley. The underlying single-thread fluvial (Unit 3) and braided fluvial (Unit 2) facies are interrupted only by limestone and bedrock outcrops at the extent of the valley. Dashed lines indicate uncertainty with question marks denoting uncertainty at depth.

A cross-valley profile generated from the thirteen cone penetrometer soundings demonstrates the presence of a uniform valley-fill sequence consisting of a 14–19 m thick layer of muds (clays and sensitive fine-grained sediments) that overlies an interlayered sequence of sand mixtures and clays before reaching consolidated silts and sands (Fig. 6b). Aside from the valley-fringes (B61-06, CPT09 & 10), each sounding presents an almost identical vertical trace with consistent and very low cone resistance within the upper mud layer (Units 4 and 5; 0.13–0.80 Mpa), a notable increase within Unit 3 (0.59–4.60 Mpa) and high, variable oscillating cone resistance typical of coarse to fine sands and coarse silts within Unit 2 (2.35–31.36 Mpa; Figs. 6b, S2 & S3). Soundings within Unit 5 are punctuated by brief, sharp increases in *q*_*t*_ at −9.5 m, most prominently within CPTs 01, 04, 05, 07 and B61-06, which is interpreted to represent a slightly coarse-grained lens of sandy silt. This corresponds to a sharp increase in grainsize and dry bulk density and a decrease in moisture content and TOC at the same depth in core Monteith-A (7,998 cal yr BP) further validating the cross-valley uniformity. Each of the ten CPTs away from the valley margins reach a much stronger, denser, underlying coarse-grained sand (Unit 2) which prevented further safe operational penetration of the cone and rods. The significant increase in material resistance due to the stiffness of the compacted sands present in the lower portions of Unit 2 prevented penetration to rock basement in most CPTs; this is a typical depth limiter of the CPT method when assessing Pleistocene-Holocene sequences^32,33^. The upper surface of this underlying consolidated sand layer varies between approximately −25 m and −19 m across the entire valley extent (Figs. 6b & S3). Limestone and underlying weathered bedrock are abruptly encountered at the margins of the valley at a depth of approximately −10 m within B61-06 and −8 m within CPTs 09 and 10 (Figs. 6b & S3).

### Depositional history - system tract identification

The basal sequence boundary presented in core Monteith-A is marked by a transition from weathered claystone to fluvial sands (Unit 1 to 2). Deposition of this unit is inferred to have commenced at approximately 20,000–18,000 yr BP contemporaneous with the last glacial maximum^3,34^. Unit 2 is interpreted to be a component of the lowstand systems tract^17^ and likely represents aggradation of braided fluvial channels, comprising medium to fine sands, in response to a rising base level in the early stages of sea-level rise (Figs. 4 & S1). This lower sand deposit is 8.5 m thick and presents its upper surface at 20.99 m depth in core Monteith-A which marks the fluvial transgressive surface and a probable shift from braided to single-thread morphology at 10,112 cal yr BP (Fig. S1)^18^. This event presents as a transition from unsorted medium sands to alternating bands of silts and silty fine sands in core Monteith-A (Unit 3; Figs. 4 & S1). This is contrasted by the identified braided to meandering morphological transition exhibited upstream within the Riverine Plain (Fig. 1)^18^, which is evidenced by an upward fining sequence, crevasse splay and point bar deposits^29^. Similar sedimentary structures are absent in core Monteith-A (Fig. S1) suggesting that the rapidly declining energy gradient within the lower Murray Gorge during the transgressive phase of the early-Holocene prevented the development of a meandering channel at our study site.

The presence of cross-lamination, shell lenses and a sharp increase in TOC marks a transitional phase with deposition of a transitional facies from 16.34 m (9,200 cal yr BP; Unit 4) in response to continued base level rise (Figs. 4 & S1). We suggest that the unique characteristics of this coastal plain system - the incredibly low gradient, discharge and sediment yield - caused a significant reduction in energy at the study site which accounts for the similarity in appearance (sediment colour, composition and texture) between Units 4 and 3, with the reduction in TOC a consequence of increased depth and salinity due to backfilling from marine flooding (Figs. 4 & S1). A transition to a laminated silt-clay sequence at 12.61 m (8,518 cal yr BP; Unit 5) marks the establishment of the mid-Holocene palaeo-Murray estuary and widespread deposition of a central basin facies (Figs. 4 & S1). The timing of transition between depositional styles correlates with numerous accounts globally of back stepping events and estuary initiation as a response to the melting of the Laurentian ice sheet (e.g.^13,27,35,36^). The laminated silt-clay sequence in Unit 5 is 11 m thick and indicates continuous uninterrupted mud laminae deposition between 8,518–5,067 cal yr BP. Finally, the radiographs suggest that the top 1.65 m of core Monteith-A may be anthropogenically disturbed which hampers interpretation in this part of the core (Fig. S1). Consequently, the transition from highstand central basin to stillstand deposits beyond 5,067 cal yr BP cannot be confidently identified.

## Discussion

**Figure 7:**
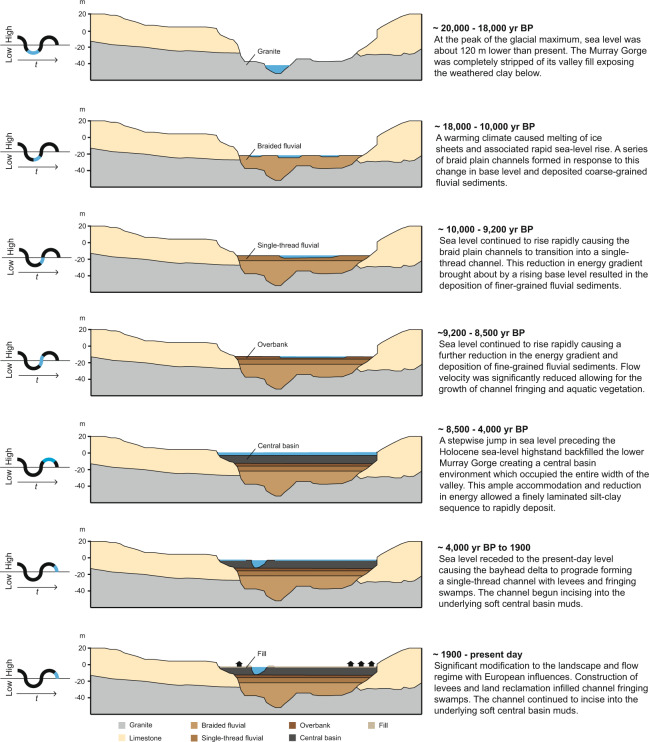
Revised geomorphologic history of the LMR. The combination of results derived from hydrodynamic models and sedimentologic data allows for the development of a revised model of sedimentary infilling of the lower Murray Gorge. These results validate and modify some aspects of Hubble and De Carli’s^65^ model, while invalidating others, and serve as a complementary upstream counterpart to the Holocene geomorphologic history of Lake Alexandrina presented by Barnett^22^.

The sedimentary facies identified in core Monteith-A record a sequence of deposition from lowstand, through transgression, to highstand. These facies demonstrate an example of the response of a stable cratonic river system to the progressive flooding of an incised valley by a rising sea level (Figs. 4 and 7)^17,20,37^. This particular presentation of Zaitlin *et al*.’s^17^ middle incised valley facies association located 104 rkm upstream of the present-day river mouth is an atypical response to sea-level rise during the Holocene. We attribute this remarkable example of inland estuarine deposition to the incredibly low relief, the fine-grained sediment load comprised almost exclusively of silt and clay, and to the large size of this semi-arid catchment. The unusually low energy of this system, indicated by modelled current velocities of <0.3 m/s (Fig. 2), enabled the formation of a condensed sedimentary section where the full transition from braided fluvial to central basin deposition occurred in approximately 1,600 years (10,112–8,518 cal yr BP). This transition is presented in core Monteith-A by an upward fining section of sands and muds (Units 3 and 4; Figs. 4 & S1). The energy available within this system was too low to produce the range of depositional conditions normally expected within the middle incised valley facies association such that a continuous condensed section is instead apparent. This has allowed for the depositional response to a full sea level transgressive cycle to be captured within the 30 m of sediment presented in core Monteith-A. These results have been summarised to produce a revised geomorphologic history of the LMR, shown in Fig. 7.

Our 3D hydrodynamic models confirm the conclusions drawn from 2D scenarios^23^ which demonstrated that, at the Holocene highstand, the Murray estuary’s central basin extended 140 rkm upstream from the present-day river mouth, well into the confines of the Murray Gorge (Fig. 2a). The 11 m thick central basin deposit presented within core Monteith-A (Fig. 4) confirms that a central basin facies was deposited at least as far upstream as Monteith (rkm 104) during the mid-Holocene (8,518–5,067 cal yr BP). The transect of CPT profiles indicate that this central basin facies was deposited across the entire width of the Murray Gorge at this location (Fig. 6b). Temporally and spatially, this sequence is contiguous with Lake Alexandrina’s central basin deposit and presents comparable mean grainsize, moisture content and TOC content to cores within the main body and palaeo-thalweg of Lake Alexandrina^21,22^. This single, continuous depositional environment in the LMR and Lake Alexandrina confirms Helfensdorfer *et al*.’s^23^ suggestion that the LMR’s Coonambidgal Formation and Lake Alexandrina’s St Kilda Formation should be considered equivalent units. The existence of this elongate estuary that was extant throughout the mid-Holocene should be considered when applying our understanding of Holocene palaeo-environments to assist in the development of management policies for the region.

In the intensively managed MDB, water use, policy and planning decisions are guided by the accuracy and quality of our knowledge of pre-anthropogenic conditions. A recent debate about the long-term variation in salinity of the Lower Lakes has been dominated by the need to maintain a freshwater supply for irrigation and environmental flows^38–41^. The Lower Lakes are held fresh by closing a series of barrages within the flood tide delta, even during periods of extreme drought (Fig. 1). The impetus to maintain these lakes as bodies of fresh water is strengthened by the Ramsar listing of the Coorong and Lakes Alexandrina and Albert wetland, which declared the waters landward of the Goolwa barrage (Fig. 1) to be fresh, and imposes an international obligation to maintain them in this condition. There are numerous, and serious, consequences of this policy, which were demonstrated during the Millennium drought (1997–2011). Water levels within the Lower Lakes dropped to a record −1.05 m Australian Height Datum (AHD; i.e. below mean sea level) exposing acid sulphate soils to a major sub-areal oxidation event as well as causing extensive property and infrastructure damage due to riverbank collapse^42,43^. Opening the barrages would have probably prevented the oxidisation event and bank failure but with the consequence of salinising the water in the Lower Lakes and downstream reaches of the LMR. The combination of our modelling results and the sedimentary sequences identified in core Monteith-A support the body of literature which suggests that the Lower Lakes were estuarine central basins subject to significant marine incursion at the Holocene highstand. A shift to a fresher water distribution within the Lower Lakes required a fall in sea level to present-day levels (Fig. 2b).

Applying the highly accurate chronology (R^2^ = 0.997) to the facies designation of core Monteith-A allows Belperio *et al*.’s^15^ sea level envelope to be constrained at the terminus of Australia’s largest river. Initially, the older limit of the envelope is supported, with marine influence first reaching Monteith (rkm 104) at 8,518 yr BP (Fig. 6a). Core Monteith-A’s sediment accumulation rate then supports a trend to the younger limit of Belperio *et al*.’s^15^ envelope over the following 2,000 years (Fig. 6a). The location and age of shells, middens, and diatom assemblages in the Murray estuary’s flood tide delta constrains the timing of sea-level stabilisation to the present-day level and the coincident shift to fresher water distribution to approximately 3,500 yr BP^4,44–46^.

Laminations that are planar in orientation, such as those deposited within the central basin of the Murray estuary, traditionally indicate that the suspended sediment settled in water that was still or almost stationary^47^. However, recent studies on the deposition of mud flocs have demonstrated that the deposition and preservation of a distinctly laminated sequence can occur at current velocities up to 0.3 m/s^24–26^. Our models demonstrate that this condition was extant over a minimum of 95% of the LMR and Murray estuary at the Holocene highstand (Fig. 2). Indeed, rhythmites or banded grainsize couplets are not uncommon in the transitional environment of the central basin of low energy estuaries and commonly record seasonal (winter/summer) variation in fluvial suspended sediment load^48–50^. We hypothesise that the Murray estuary’s central basin deposit comprises such couplets as the present-day flow (which is dominantly derived from the Murray sub-catchment) is distinctly seasonal due to the annual spring/summer melting of the winter snow pack that develops on the Australian Alps. Evidence for such a pronounced seasonality within the LMR during the mid- to late-Holocene has previously been described through fluctuating palaeo-salinities identified in fish otoliths located immediately upstream from our study site (rkm 125–110)^51^. This hypothesis forms the basis of a follow-up study which, if confirmed, may enable the extraction of a record of mid- to late-Holocene annual climatic variability for southeastern Australia from core Monteith-A.

The Holocene provides a useful analogue of potential future change to assist in the adaptation of natural resource management policies to the changing climate and rising sea level. Variation in the Holocene palaeo-climate of the MDB has been inferred from sedimentary analyses of two marine sediment cores collected from the Lacepede Shelf offshore from the Murray Mouth (MD03-2611 and MD03-2607)^6,11^. Several previous studies have used these high resolution, continuous sedimentary records, located at the terminus of the MDB, to constrain climatic conditions for the MDB during the Holocene^2^. Climatic interpretation of cores MD03-2611 and MD03-2607 rely on the clay/silt-ratio, as a proxy for fluvial/aeolian terrigenous input, and illite concentration, as a proxy for sediments derived from the Murray sub-catchment (Fig. 4)^6,11^. However, our study demonstrates that a large natural sediment trap was extant in the lower Murray Gorge and Lake Alexandrina for much of the Holocene where the extensive central basin intercepted and likely prevented the delivery of terrigenous sediment derived from the MDB to the Southern Ocean and Lacepede Shelf. Other studies have demonstrated that the significant accommodation space provided by coastal plain estuaries, particularly during initial stages of estuarine development in the early- to mid-Holocene, can result in up to 95% of fluvial sediment being prevented from reaching the continental shelf^52,53^. The LMR is an extreme end-member example of this phenomenon as this coastal plain river presents a particularly low gradient which significantly limits stream power^54^. It follows therefore that there must have been significant terrigenous sediment sequestration within the Holocene sedimentary record of the young estuary–which our results demonstrate–and a consequential reduction in the delivery of terrigenous sediment and deposition in the sedimentary record immediately offshore.

Given the results we have presented here, we suggest an alternate and more plausible interpretation to the palaeo-climate signal presented in Gingele *et al*.^6,11^. We suggest that the transitional point marked by a sudden decrease in the clay/silt-ratio and a trend to decreasing illite concentrations from 8,500–8,300 yr BP (Fig. 4)^6,11^ is better explained by the inception and widespread development of the Murray estuary. This development of the central basin and estuary within the lower Murray Gorge is another example of the response of a large incised-valley river system to the melting of the Laurentian ice sheet and associated sudden increase in sea level between 8,500–8,200 yr BP that initiated estuaries globally^13,27,35^. This elongate central basin afforded ample accommodation space for the sequestration of terrigenous sediments within the LMR and Lower Lakes. The timing of reduced clay/silt-ratios in MD03-2607 corresponds exactly with the transition from fluvial to estuarine deposits in core Monteith-A and is also contemporaneous with the commencement of deposition of the central basin laminated silt-clay sequence at 8,518 cal yr BP (Figs. 4 and 6a). Gingele *et al*.’s^6,11^ continued trend of apparent increasing aridity cumulating in the commencement of arid conditions from 5,500 yr BP corresponds to the rapid deposition of an 11 m uninterrupted laminated sequence spanning from 8,518–5,067 cal yr BP in core Monteith-A (Fig. 4). Similarly, the sharp decrease in clay/silt-ratio and illite concentration that marks a transitional point in the sedimentary record within marine cores MD03-2607 and MD03-2611^6,11^ corresponds to the Holocene sea-level highstand of +2 m between 7,000–6,000 yr BP^15,16^. At this time, the incursion of central basin conditions into the lower Murray Gorge was at its maximum and likely presented over a 100 rkm section of Lake Alexandrina and the lower Murray Gorge to trap sediment (Fig. 4).

This study demonstrates the response of an extreme end-member (low discharge, low sediment load, low gradient, and large catchment) coastal plain system to a rising sea level during the Holocene, revealing valley-wide sequences from lowland, through transgression to highstand (Fig. 6). We adopt a novel multidisciplinary approach to assessing palaeo-environmental change by independently verifying best-estimate Holocene highstand and late-Holocene 3D hydrodynamic models with sedimentary analyses, an approach which, to date, has not been conducted. Our results confirm and extend the conclusions of Helfensdorfer *et al*^23^. and show that the Holocene sea-level highstand induced an extensive estuarine environment in the LMR, driving the tidal and brackish limits well upstream into the Murray Gorge (Fig. 2). This is an atypical, extreme end-member example of inland estuarine deposition whereby estuarine facies successions are presented well within the typically riverine portion of the coastal plain system rather than restricted to within or immediately upstream of the stilling basin of the barrier estuary (in this case Lake Alexandrina).

These results support the contention that the Lower Lakes developed as part of the Murray estuary and therefore could not have been freshwater bodies for the duration of the Holocene. We demonstrate that the mid-Holocene sea-level highstand created a single, vast central basin environment throughout the Lower Lakes and upstream to Wall Flat (rkm 140, Fig. 2), that was characterised by a finely laminated silt-clay sequence, as previously described within Lake Alexandrina by Barnett^21,22^. A 30 m sediment core (Monteith-A) and transect of CPT soundings independently verify the modelling in demonstrating that this deposit is valley-wide throughout the lower reaches of the LMR. This is consistent with previous accounts of the upper valley-fill as comprising the Coonambidgal Formation muds without evidence of coarse-grained channel sediments^18^. The presence of an uninterrupted, valley-wide 11 m central basin deposit (8,518–5,067 cal yr BP) suggests that the rising sea level would have strongly supressed deposition of terrigenous sediment on the Lacepede Shelf and may have prevented the offshore delivery of terrestrial sediment entirely. Instead, this sediment was trapped within the young estuary and deposited in discrete laminations within the Lake Alexandrina and the lower reaches of the Murray Gorge. This finding suggests that palaeo-climatic inferences drawn from the terrigenous flux signal within marine cores MD03-2611 and MD03-2607 presented elsewhere may not be valid and that Holocene palaeo-climatic reconstructions which rely on conclusions drawn from the Lacepede Shelf cores must be re-evaluated and reconsidered. Further investigation of the record of sedimentation preserved in core Monteith-A has the potential to provide a detailed, reliable, high-resolution palaeo-climate signal for the MDB during the mid-Holocene.

## Methodology

### Hydrodynamic model

Best-estimate Holocene highstand and late-Holocene models from Helfensdorfer *et al.*^23^ were replicated in 3D using TUFLOW FV, a finite volume numerical model. From the conclusions of Helfensdorfer *et al.*^23^ the most appropriate scenarios that adequately captured the transition in palaeo-environmental character from the Holocene highstand to the pre-anthropogenic condition required a change in bathymetric surface and sea level only. The best-estimate Holocene highstand scenario adopts an inferred mid-Holocene bathymetry (S_mid_) with modern-day barrier morphology (B_mod_)^23^. The average discharge prior to anthropogenic modification of the flow regime (D_av_) is applied at Blanchetown (rkm 282), while +2 m is superimposed on a modern-day tidal dataset to represent sea level at the Holocene highstand (WL_2_)^15,16,23^. Together, this set of variables corresponds to Helfensdorfer *et al*.’s^23^ scenario S_mid_WL_2_D_av_B_mod_. The corresponding late-Holocene scenario, depicting the natural system prior to anthropogenic alteration to the flow regime, differs only by the adoption of pre-regulation bathymetry (S_up_) and a modern-day tidal dataset at present-day sea level (WL_0_), corresponding to Helfensdorfer *et al.*’s^23^ scenario S_up_WL_0_D_av_B_mod_.

The 3D models presented here used hybrid z-sigma coordinates with a total of 8 vertical layers–6 z-layers and 2 surface sigma layers–and a second order vertical solution, parametric vertical mixing model, and density coupled salinity. All other aspects of the model set up were held constant to those of Helfensdorfer *et al*.^23^. Details on the numerical model set up, morphology and sensitivity testing are given in Helfensdorfer *et al*.^23^.

Under present day conditions, with artificially high lake levels and supressed marine influence, wind-waves are the primary driver of sediment resuspension within the Lower Lakes^55^. The temporally and spatially extensive deposition of a laminated sequence throughout the main body of Lake Alexandrina during the mid- to late-Holocene^21,22^ suggests that salinity-assisted flocculation sufficiently counteracted the influence of wind-waves. Our results support this contention suggesting that the Lower Lakes were subject to significant marine influence at the Holocene highstand. The presence of a salt wedge at depth would have assisted floc formation and settling of fine suspended sediment^56^. Previous studies into the influence of wind-generated waves on saline incursion have demonstrated that the dominant south-westerly wind direction results in wind-waves driving backflow events into the LMR^57^. With wind-waves absent from our model, modelled saline incursion results reflect calm conditions and could therefore be considered conservative estimates when compared to likely conditions under the dominant wind regime.

### Fieldwork

Monteith was chosen as the most suitable fieldwork site along the LMR as sensitivity testing of hydrodynamic models by Helfensdorfer *et al*^23^. indicated that this location marks the minimum upstream extent of the Murray estuary’s central basin at the Holocene highstand. The study site, located at rkm 104, is situated within the Monteith Irrigation Management Zone and, as such, has been subject to artificial levee construction, land reclamation and laser levelling allowing access to naturally inundated land. For this study, ten CPTs were collected in a transect perpendicular to the channel at 100 m spacing, commencing 100 m from the left bank, using a 22 t 6 × 6 specialist CPT truck. To provide a whole-of-valley analysis, results of three CPTs taken on the opposite side of the channel at 100 m spacing, and employing the same specialist CPT truck, were acquired for analysis. Situated within 1 km of horizontal continuity from the CPTs collected in this study, the use of these additional three CPTs to complete the transect was deemed appropriate given the 282 rkm longitudinal extent of the study area. For each of the thirteen CPTs, cone resistance (*q*_*c*_), sleeve friction (*f*_*s*_), dynamic pore pressure (*u*_*2*_), inclination (*I*), friction ratio (*R*_*f*_) and corrected cone resistance (*q*_*t*_) were collected at 1 cm resolution in real time. The 30 m sediment core, Monteith-A, was taken at the location of CPT08 (Fig. 1) using a Commachio MC900 Multi-Sonic drilling rig, split at the time of extrusion into 1 m sections and placed immediately into cold storage.

### Chronology

Five samples of >63 µm charcoal fragments and 4 samples of fibrous organic fragment were submitted for accelerator mass spectrometry (AMS) ^14^C radiocarbon dating (lab ID: UBA) and calibrated using Calib 7.0.4 applying the SHCal13 calibration curve^58^. An age-depth model was developed in R using Bacon 2.3.3^59^. Assuming the surface to be modern, there is a significantly different sediment accumulation rate above the youngest age, which is unrealistic particularly given the potential of disturbance of the near surface due to agricultural activities. To account for this, a hiatus was input into the model directly above the shallowest ^14^C date at 2.10 m.

### Sedimentary analyses

Core Monteith-A was subsampled at 10 cm resolution for sedimentary analyses. Grainsize samples were subject to 35% H_2_O_2_ to oxidise the organic material, then disaggregated by adding hexametaphosphate 50 g/L and rotating samples for 12 hours prior to analysis. Grainsize analyses were conducted using a Malvern Mastersizer 2000 and statistical analyses conducted using GRADISTAT 8.0^60^ adopting the classification scheme of Folk and Ward^61^. Moisture content, unit weight and bulk density were assessed by subsampling sediment into rings of a known weight and volume and oven drying overnight at 60 °C. Subsequently, these dried samples were crushed and placed into a furnace at 550 °C to ascertain a crude measure of the organic content of the sediment through loss on ignition (LOI).

### Interpretation of cone penetrometer soundings

The high resolution, fast testing rate and low cost makes the CPT a novel and desirable method of interpreting sequence stratigraphy at a site^32,33^. The use of CPT data to infer sequence stratigraphy is particularly robust when calibrated against a sediment core taken at the same or adjacent location. CPT08 was calibrated against the particle size distribution of core Monteith-A to assess the suitability of extrapolating results to infer sedimentary units from the thirteen CPTs obtained across the valley. The two key parameters are: the cone tip resistance (*q*_*c*_), which is considered indicative of the density and consistency of the sediment; and, the friction ratio (*R*_*f*_), which is considered indicative of sediment grainsize and texture^28,32,33^. Together these parameters are plotted using Robertson’s^28^ *q*_*c*_/*R*_*f*_ classification chart to determine the SBT.

Previous studies which have used CPT soundings to determine estuarine stratigraphy typically attribute the bounds and average *q*_*c*_/*R*_*f*_ values of each sediment facies in the calibrated CPT to the soundings across the valley to develop a whole-of-valley analysis (e.g.^32,33,62,63^). Here, we enhance this methodology by adopting a k-medoids clustering analysis^30^ in Matlab on the calibrated CPT soundings (CPT08). The 1 cm resolution soundings were averaged in 10 cm intervals so as to be directly comparable to the grainsize sampling resolution of core Monteith-A. Due to the incredibly low strength of the Coonambidgal Formation muds, negative friction values were recorded within CPTs B61-02, B61-04 and CPT05; these were excluded from the analysis. A k-medoids clustering analysis adopting a squared Euclidean distance metric was performed on log_10_ transformed *q*_*c*_*/R*_*f*_ data for CPT08 (and cross referenced relative to depth, to assess whether the facies divisions identified in core Monteith-A were correctly captured through the SBT analysis). The elbow and average silhouette methods were adopted to determine the optimal number of clusters. Both approaches suggested two clusters was optimal, which differentiated the Monoman Formation sands and the Coonambidgal Formation muds. This was, however, insufficient for the identification of sequence stratigraphy. Both methods returned six clusters as the second most optimal number of clusters. Each of the remaining twelve CPT soundings were then assessed in turn by attributing each individual soundings to the nearest medoid of each of the six clusters identified in CPT08 also using a squared Euclidean distance measure. The results of this clustering analysis were then plotted relative to depth and distance across the valley to infer sequence stratigraphy (refer to supplementary materials).

## Supplementary information

Supplementary materials.

## Data Availability

The data sets generated and/or analysed during this study are available from the corresponding author on reasonable request.
