# Supplementary material for: Atypical responses of a large catchment river to the Holocene sea-level highstand: The Murray River, Australia
Source: Sci Rep. 2020 May 5;10:7503. doi: 10.1038/s41598-020-61800-x (PMC7200661; doi:10.1038/s41598-020-61800-x)
Supplement: Supplementary file 1 — Supplementary materials. [file 41598_2020_61800_MOESM1_ESM.pdf]

SUPPLEMENTARY MATERIALS

Atypical responses of a large catchment river to the Holocene sea-level highstand: The Murray River, Australia

Authors: Anna M. Helfensdorfer[1,2]*, Hannah E. Power[2], Thomas C.T. Hubble[1]

Affiliations:

1. School of Geosciences, The University of Sydney, Sydney, NSW 2006, Australia

2. School of Environmental and Life Sciences, The University of Newcastle, Callaghan, NSW 2308, Australia

*Correspondence should be addressed to A.M.H. (email: anna.helfensdorfer@sydney.edu.au)

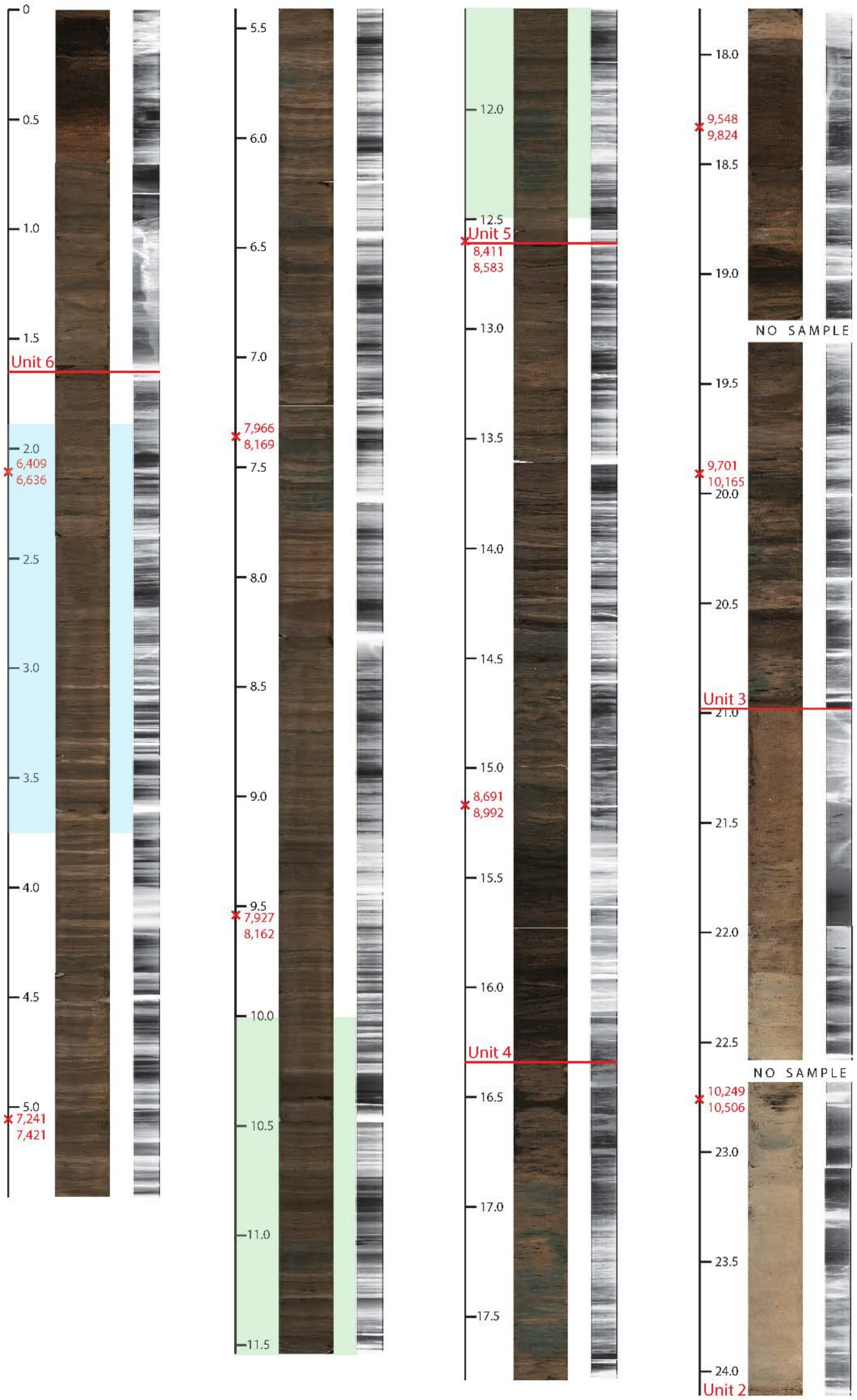

Figure S1: Core Monteith-A imagery. Optical imagery and radiographs of 24.12 m of core Monteith-A showing locations of ^{14}C dates and calibrated ages (in cal yr BP). Unit boundaries (red), worldwide estuary initiation event (green) and sea-level highstand (blue) are given as per Figure 4. Scale is depth in meters.

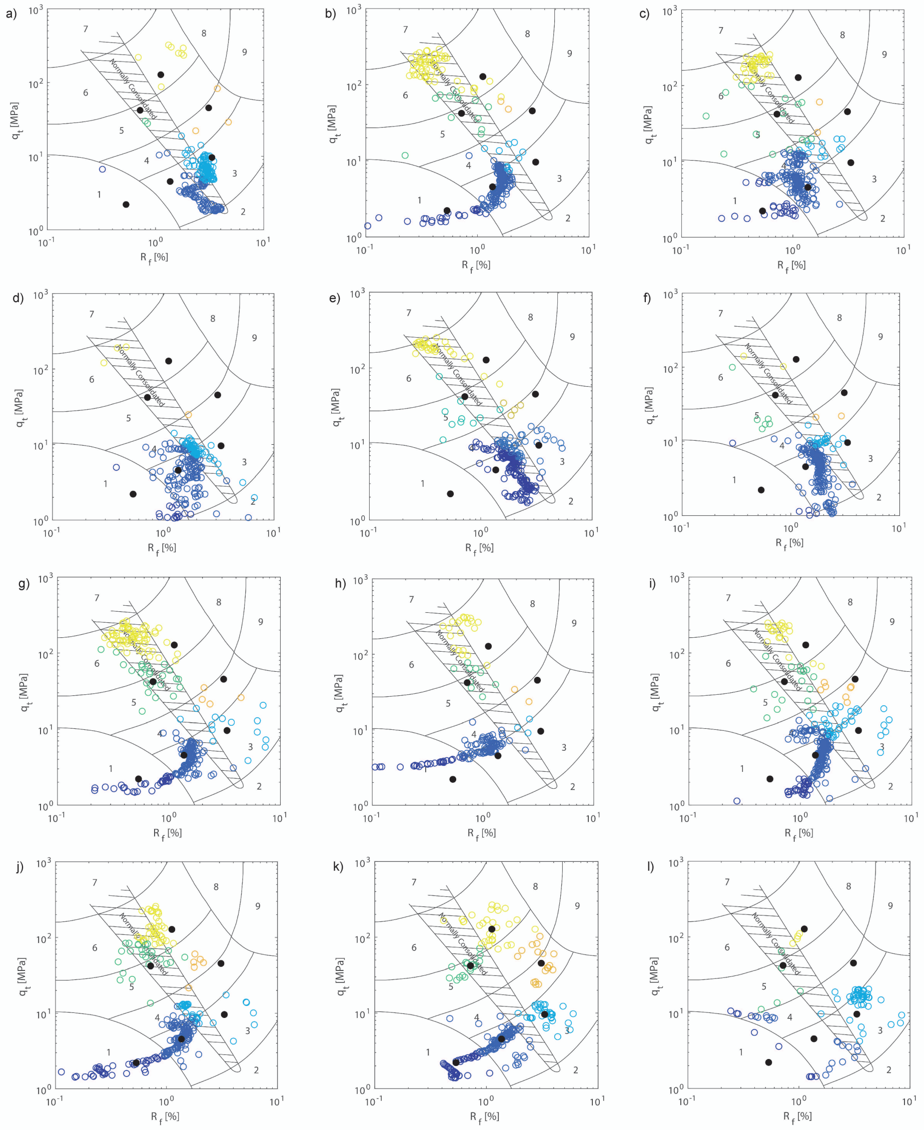

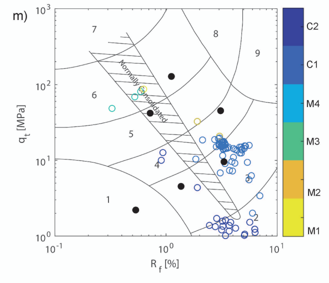

n)

Zone	Soil behaviour type
1	Sensitive fine-grained
2	Clay - organic soil
3	Clays - clay to silty clay
4	Silt mixtures - clayey silt to silty clay
5	Sand mixtures - silty sand to sandy silt
6	Sands - clean sand to silty sand
7	Dense sand to gravelly sand
8	Stiff sand to clayey sand*
9	Stiff fine-grained*

*overconsolidated or cemented

Figure S2: q_c/R_f plots for each CPT giving Robertson's (*28*) SBT coloured by cluster relative to the six clusters identified within CPT08. (**a**) B61-06; (**b**) B61-02; (**c**) B61-04; (**d**) CPT01; (**e**) CPT02; (**f**) CPT03; (**g**) CPT04; (**h**) CPT05; (**i**) CPT06; (**j**) CPT07; (**k**) CPT08; (**l**) CPT09; (**m**) CPT10; (**n**) description of soil behaviour type given by each of Robertson's (*28*) nine zones. Black points denote the medoid of each cluster identified for CPT08, with the cluster legend given in panel (**m**). Clusters M1-M4 comprise sediments of the Monoman Formation, while clusters C1-C2 comprise sediments of the Coonambidgal Formation.

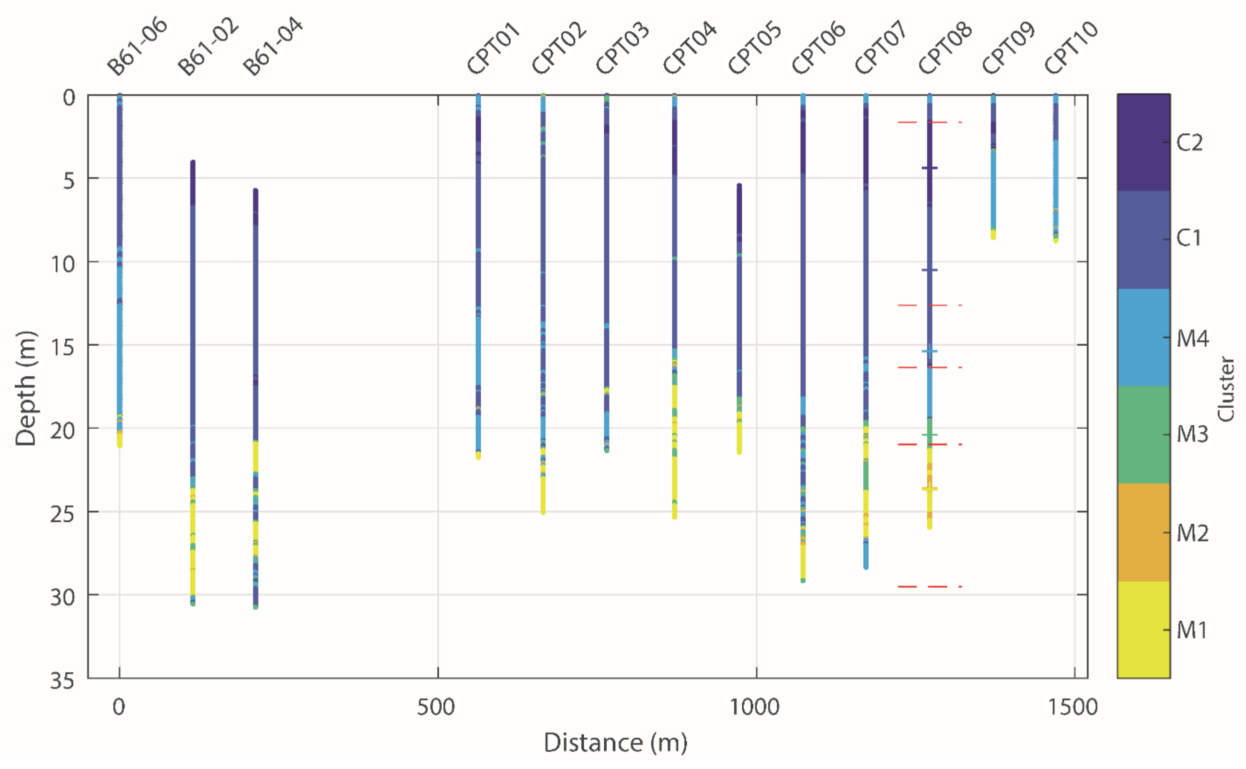

Figure S3: CPT cluster analysis by depth. Plotting the results of individual clustering analyses (Fig. S1) by depth and relative position across the valley reveals vertical and lateral trends in sediment geotechnical properties. The median depth of each cluster in CPT08 is denoted by a cross, with the red hatched line indicating the depth of each sedimentary unit identified within core Monteith-A. Negative sleeve friction values prevented q_c/R_f analysis for the top few meters of sediment within B61-02, B61-04 and CPT05. Data displayed here was used to inform the creation of the cross section in Figure 6b.
